# Dynamics of Order Reconstruction in a Nanoconfined Nematic Liquid Crystal with a Topological Defect

**DOI:** 10.3390/ijms141224135

**Published:** 2013-12-12

**Authors:** Xuan Zhou, Zhidong Zhang

**Affiliations:** Department of physics, Hebei University of Technology, Tianjin 300401, China; E-Mail: zhouxuan198536@163.com

**Keywords:** biaxial transition, topological defect, eigenvalue exchange, response time

## Abstract

At the wall in a hybrid nematic cell with strong anchoring, the nematic director is parallel to one wall and perpendicular to the other. Within the Landau-de Gennes theory, we have investigated the dynamics of *s* = ±1/2 wedge disclinations in such a cell, using the two-dimensional finite-difference iterative method. Our results show that with the cell gap decreasing, the core of the defect explodes, and the biaxiality propagates inside the cell. At a critical value of *d*_c_* ≈ 9ξ (where ξ is the characteristic length for order-parameter changes), the exchange solution is stable, while the defect core solution becomes metastable. Comparing to the case with no initial disclination, the value at which the exchange solution becomes stable increases relatively. At a critical separation of *d*_c_ ≈ 6ξ, the system undergoes a structural transition, and the defect core merges into a biaxial layer with large biaxiality. For weak anchoring boundary conditions, a similar structural transition takes place at a relative lower critical value. Because of the weakened frustration, the asymmetric boundary conditions repel the defect to the weak anchoring boundary and have a relatively lower critical value of *d*_a_, where the shape of the defect deforms. Further, the response time between two very close cell gaps is about tens of microseconds, and the response becomes slower as the defect explodes.

## 1. Introduction

Topological defects arise as a result of broken continuous symmetry and are ubiquitous in nature, from microscopic condensed matter systems governed by quantum mechanics to a universe in which gravity plays a decisive role [1–3]. Defects in liquid crystals (LCs) have been the subject of much interest, still offering unsolved problems. Commonly observed defects in the uniaxial nematic phase are typically point defects with topological charge *s* = 1 and line defects with topological charge *s* = ±1/2 [4]. There are two types of *s* = ±1/2 disclination lines [5]. The first type is wedge disclination, with the rotation vector parallel to the disclination line, while the second is twist disclination, with the rotation vector perpendicular to the disclination line. The region where the presence of a defect causes apparent deviations from bulk ordering is referred to as the defect core [6].

Eigenvalue exchange (also called order reconstruction) was first shown by Schopohl and Sluckin within the core of *s* = ±1/2 wedge disclinations [7] and has aroused numerous subsequent studies on the detailed core structure in nematic LCs for various boundary conditions theoretically [8–13] and experimentally [14–17]. In particular, Palffy-Muhoray [8] investigated the eigenvalue exchange problem in a hybrid alignment nematic (HAN) cell with perpendicular alignment of NLC molecules on one surface and parallel alignment on the other. A structure transition from the eigenvector rotation configuration to the eigenvalue exchange is expected when the cell gap is decreased. Similar results are achieved in a twisted nematic (TN) cell with π/2 boundary twist configuration [9].

Recently, the atomic force microscope (AFM) has been used as a unique tool to measure the separation dependence of surface forces with unprecedented accuracy and flexibility [18–26]. This is not only important for studying the molecular and surface forces due to their potential application in self-assembly at the nanoscale, but is also important for the structural analysis of the interfaces of soft matter and complex fluids in contact with a solid wall. As an example, the structure of LC layers have been successfully analyzed using the force spectroscopy mode of an AFM [18–20]. Particularly, Carbone *et al*. [18] give the experimental evidence of the eigenvalue exchange in a thin HAN layer in the presence of a topological defect. We know that the speed of the AFM probe is about 1–5 μm/s, which is much slower. corresponding to the dynamic response of the structural transition (~10–100 μs) [14–16], in other words, the speed of the AFM probe is slow enough for the system to undergo a structural transition; so, the AFM can be used to investigate the dynamic process of the structural transition as the call gap varies.

Recently, many theoretical investigations have focused on the dynamical behavior of structure transition, using the *Q* tensor numerical model [27–31], which also describe the effect of order reconstruction-eigenvalue exchange. In this study, we will investigate the dynamics of biaxial transition in a nanoconfined HAN layer with a topological defect numerically, using the two-dimensional finite-difference iterative method. Our study is based on the Landau-de Gennes theory [32] describing the orientational order of an LC in terms of a second-rank tensor *Q*, which encompasses both uniaxial and biaxial states.

## 2. Theoretical Basis

### 2.1. Free Energy

Our theoretical argument is based on the Landau-de Gennes theory [32], in which the orientational order of an LC is described by a second-rank symmetric and traceless tensor [33]:

(1) $$Q=\sum_{i=1}^{3}\lambda_{i}e_{i}⊗e_{i}.$$

Here, λ_i_ and *e*_i_ are the *i*_th_ eigenvalue and the *i*_th_ eigenvector of *Q*, respectively. In the isotropic phase, *Q* vanishes. In the uniaxial ordering, *Q* has two degenerate eigenvalues and can be represented by

(2) $$Q=S(3\vec{n}⊗\vec{n}-\text{I}),$$

where *S* is the uniaxial scalar parameter, and the unit vector, *n⃑*, is the nematic director pointing along the local uniaxial ordering direction. In Equation (2), *S*can have either sign: when it is positive, the ensemble of molecules represented by *Q* tends to be aligned along *n⃑*, whereas when *S* is negative, it tends to lie in the plane orthogonal to *n⃑*.

Finally, when all eigenvalues of *Q* are distinct, the LC is in a biaxial state. The degree of biaxiality is measured by the biaxiality parameter, β^2^, defined as [34]

(3) $$\beta^{2}=1-\frac{6{\left[tr\left(Q^{3}\right)\right]}^{2}}{{\left[tr\left(Q^{2}\right)\right]}^{3}},$$

which is a convenient parameter for illustrating spatial inhomogeneities of *Q* and ranges in the interval [0, 1]. In all uniaxial states with two degenerate eigenvalues, β^2^ = 0, while states with maximal biaxiality correspond to β^2^ = 1. Since *tr*(*Q*^3^) = 3det*Q*, the states with β^2^ = 1 are precisely those where det*Q* = 0, which further implies that at least one eigenvalue of *Q* vanishes.

Following the notation in [7], the Landau-de Gennes free energy density of LC is given by *f* = *f*_bulk_{*Q*_αβ_} + *f*_e_{*Q*_αβ_, ▽}, in which

(4) $$f_{\text{bulk}}=AtrQ^{2}+\frac{2}{3}BtrQ^{3}+\frac{1}{2}C{\left(trQ^{2}\right)}^{2}$$

is the bulk energy that describes a homogeneous phase. In *f*_bulk_, *B* and *C* are positive constants, and *A* is assumed to vary with temperature *T* in the form *A* = *a* (*T* − T*), where *a* is a positive constants and T* is the nematic supercooling temperature. Equation (4) gives the bulk equilibrium value of the uniaxial scalar order parameter in Equation (2), $s_{eq}=-\frac{B}{12C}(1+\sqrt{1-\frac{24AC}{B^{2}}})$, which also depends on the temperature.

The free-energy, *f*_e_, which penalizes gradients in the tensor order parameter field, is given in the form

(5) $$f_{e}=L_{1}\frac{\partial Q_{ij}}{\partial x_{k}}\frac{\partial Q_{ij}}{\partial x_{k}}+L_{2}\frac{\partial Q_{ij}}{\partial x_{j}}\frac{\partial Q_{ik}}{\partial x_{k}}+L_{3}\frac{\partial Q_{ij}}{\partial x_{k}}\frac{\partial Q_{ik}}{\partial x_{j}}.$$

The coefficients, *L*_i_, are related to the splay, twist and bend elastic constants. In this work, we use the one-elastic-constant approximation just for simplicity, where the splay, twist and bend elastic constants have a common value that depends quadratically on the scalar order parameter *K* = 2*S*^2^*L*_1_, and Equation (5) reduces to

(6) $$f_{e}=L_{1}\frac{\partial Q_{ij}}{\partial x_{k}}\frac{\partial Q_{ij}}{\partial x_{k}}.$$

The boundary conditions at the LC interfaces are taken into account by the surface term, *f*_s_, describing the interaction between the nematic molecules close to the substrate and the substrate itself, which is given by

(7) $$f_{s}=\frac{W_{s}}{2}tr{\left(Q-Q_{s}\right)}^{2},$$

where *W*_s_ = *W/S* is the anchoring strength, with *W* being the anchoring strength in the Frank elastic theory, and *Q*_s_ is the value of the tensor order parameter preferred by the surface [27]. The stable solution of *Q* on the boundary condition must satisfy the Neumann condition

(8) $$\frac{\partial f_{s}}{\partial Q_{ij}}+\nu_{k}\frac{\partial f}{\partial Q_{ij,k}}=0,$$

where *ν*_k_ is the *k*_th_ component of the outward vector normal to the substrate. This expression allows us to impose on the surface substrate uniaxial, as well as biaxial conditions. For rigid anchoring, this is equivalent to imposing the Dirichlet condition on the surface, *i.e.*,

(9) $$Q=Q_{s}.$$

After an appropriate rescaling of the variables, we can simplify the calculating process. Here, we follow the rescaling of Schopohl and Sluckin [7] by defining the following dimensionless quantities: *f̃* ≡ *f*/[*B*^4^ / (9*C*)^3^], $\tilde{Q_{ij}}=Q_{ij}/q_{0}$, *d̃* ≡ *d*/*ξ*, where $q_{0}=-\frac{B}{9C}$ is the order parameter at the isotropic-nematic phase transition point and $\xi =\sqrt{\frac{L_{1}}{-Bq_{0}}}=\sqrt{\frac{9CL_{1}}{B^{2}}}$ is the characteristic length for order-parameter changes; then, the rescaled form of Equations (4)–(7) can be written as

(10) $${\tilde{f}}_{\text{bulk}}=\tilde{A}tr{\tilde{Q}}^{2}-\frac{2}{3}tr{\tilde{Q}}^{3}+\frac{1}{9}{\left(tr{\tilde{Q}}^{2}\right)}^{2},$$

(11) $${\tilde{f}}_{e}=\frac{\partial {\tilde{Q}}_{ij}}{\partial {\tilde{x}}_{k}}\frac{\partial {\tilde{Q}}_{ij}}{\partial {\tilde{x}}_{k}},$$

(12) $${\tilde{f}}_{s}=\frac{{\tilde{W}}_{s}}{2}\xi tr{\left(\tilde{Q}-{\tilde{Q}}_{s}\right)}^{2},$$

where the rescaled parameter, $\tilde{A}=\frac{9AC}{B^{2}}$, defines the temperature scale and ${\tilde{W}}_{S}=\frac{W_{S}}{W_{0}}$, with *W*_0_ = −*q*_0_*Bξ*. We notice that the isotropic-nematic transition takes place at $\tilde{A_{0}}=\frac{1}{3}$.

In the scaling employed above, the rescaled uniaxial ordering has the form

(13) $$\tilde{Q}=\tilde{S}(3\vec{n}⊗\vec{n}-\text{I}),$$

where $\tilde{S}=\frac{S_{eq}}{q_{0}}=\frac{3}{4}(1+\sqrt{1+\frac{8\tilde{A}}{3}})$ is the rescaled uniaxial scalar parameter at equilibrium.

### 2.2. Geometry of the Problem

**Figure 1.**
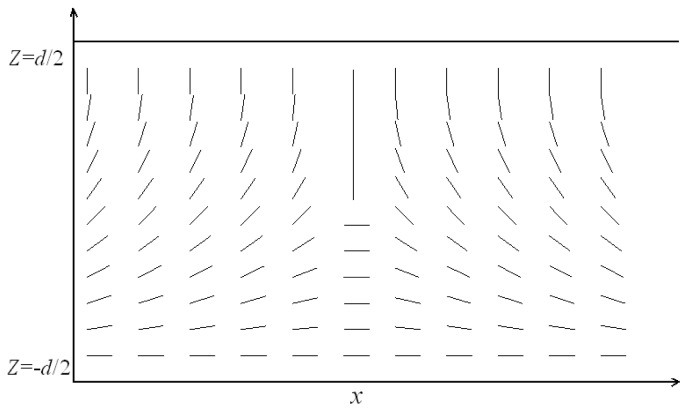
The geometry of the problem.

We choose a HAN cell with strong anchoring boundary conditions; the nematic director is parallel to one wall and perpendicular to the other (see Figure 1). The model has two stable degenerate configurations with a rotation difference of π. The plates are placed at *z* = ±*d*/2 of a Cartesian coordinate system. The lengths, *d*_x_ and *d*_y_, of the cell along the *x*- and the *y*-axes are much larger than *d* (*d_x_* ~ *d_y_* >> *d*).

We study the structure of *s* = ±1/2 wedge disclinations. The disclination line is parallel to the *y*-axis of the simulation cells, and we seek solutions independent of *y*.

At the two plates *z* = ±*d*/2, we enforce the uniaxial anchoring represented by *ϕ*_−_*_d_*_/2_*=* 0 and *ϕ*_+_*_d_*_/2_ = π/2, that is

(14) $${\tilde{Q}}_{-d/2}=\tilde{S}\left(\begin{array}{ccc} 2 & 0 & 0\\ 0 & -1 & 0\\ 0 & 0 & -1 \end{array}\right),$$

(15) $${\tilde{Q}}_{+d/2}=\tilde{S}\left(\begin{array}{ccc} -1 & 0 & 0\\ 0 & -1 & 0\\ 0 & 0 & 2 \end{array}\right).$$

On the lateral walls at *x* = ±*d_x_*/2, we also prescribe fixed boundary conditions with uniaxial ordering. The total rotation at *x* = *d_x_*/2 is −π/2, while at *x* = −*d_x_*/2 is *π*/2. These boundary conditions are compatible with the generation of line defects with topological charge *s* = −1/2, with which we shall also apply to line defects with topological charge *s* = 1/2, with the conditions at the lateral walls exchanged.

### 2.3. Numerical Methods

We compute the evolution of the LC with a dynamic theory for its tensor order-parameter field, *Q*(*r*,*t*). The local values of the scalar order parameter, *S*, and the director, *n⃑*, can be obtained from *Q* through its largest eigenvalue and its associated eigenvector, respectively. According to [17], the evolution equation describing the dynamics of *Q* can be written as

(16) $$\frac{\partial Q}{\partial t}=\Gamma \left[-\frac{\delta f}{\delta Q}+\frac{1}{3}tr\left(\frac{\delta f}{\delta Q}\right)\text{I}\right].$$

The coefficient, *Γ*, is given by *Γ* = 6D*/[1 − 3*tr*(*Q*^2^)]^2^, where D* is the rotational diffusion for the nematic. In these equations, it is assumed that *δf*/*δQ* has been symmetrized. The stable solution of *Q* must satisfy the system Equations (16) and, on the boundary, the Neumann condition (Equation (8)) or Dirichlet condition (Equation (9)).

The numerical calculations are to be intended with respect to the scaled variables. When the functional derivatives in Equation (16) are evaluated, one obtains a partial differential equation for *Q̃* in the bulk,

(17) $$\frac{\partial \tilde{Q}}{\partial t}=-\tilde{\Gamma }\left\{2\tilde{A}\tilde{Q}+2{\tilde{Q}}^{2}+\frac{2}{9}tr({\tilde{Q}}^{2})\left(\tilde{Q}+3\text{I}\right)-2{\tilde{\nabla }}^{2}\tilde{Q}\right\},$$

with the boundary conditions,

(18) $${\tilde{\nabla }}_{z}\tilde{Q}(-d/2)=\frac{{\tilde{W}}_{s}}{2}\left[\tilde{Q}(-d/2)-{\tilde{Q}}_{s}(-d/2)\right],$$

(19) $${\tilde{\nabla }}_{z}\tilde{Q}(-d/2)=\frac{{\tilde{W}}_{s}}{2}\left[\tilde{Q}(-d/2)-{\tilde{Q}}_{s}(d/2)\right],$$

where Γ̃ =Γ × (−*Bq*_0_) and $\tilde{\nabla_{z}}=\frac{\partial }{\partial \tilde{Z}}$. In general, Equation (17), subject to boundaries Equations (14) and (15), has more than one solution. One is that the structure satisfies the initial conditions given above, *i.e.*, the configuration with a line defect; the second one is a pure HAN configuration consisting of a rotation of the eigenvector of *Q* (the director) with the *z*-axis only, *i.e.*, a HAN configuration with no defect; and the third is the configuration with a biaxial layer, where there is little or no net rotation of the eigenvector, *i.e.*, eigenvalue exchange configuration. In this work, we focus on the equilibrium configuration and dynamics with the initial conditions given in Section 2.2.

We use a two-dimensional finite-difference iterative method employed in our previous studies in [35]. To discretize Equation (17), we replace the derivatives with finite difference as follows:

(20) $$\left[\tilde{Q}(t+\Delta t)-\tilde{Q}(t)\right]/\Delta t=-\tilde{\Gamma }\left\{2\tilde{A}\tilde{Q}(t)+2{\tilde{Q}}^{2}(t)+\frac{2}{9}tr{\tilde{Q}}^{2}(t)\left(\tilde{Q}(t)+3\text{I}\right)-2{\tilde{\nabla }}^{2}\tilde{Q}(t)\right\}.$$

In our numerical calculations, we have found that a discretization with a time step given by 4 × 10^−10^ is sufficient to guarantee the stability of the numerical procedure. In addition, our equilibration runs take 10^6^, which has been confirmed as sufficient for the system to reach equilibrium state.

We let the system relax from an initial condition under the boundary conditions given in Section 2.2. We have calculated the tensor *Q* after the system to reach equilibrium state, and then, the current tensor is diagonalized. Accordingly, the director is identified by the eigenvector possessing the largest eigenvalue. Then, the degree of biaxiality is measured by the biaxiality parameter, β^2^, given by Equation (3), and the corresponding free energy is calculated. When the equilibrium state with a different cell gap is reached, we compute the dynamic evolution process between different cell gaps with the evolution equation given by Equation (16).

## 3. Results

In this section, we present our numerical results. According to the parameters given in [36], we have *a* = 0.043 × 10^−6^ J/m^3^, *B* = −1.06 J/m^3^, *C* = 0.87 J/m^3^, *L*_1_ = 2.25 × 10^−12^ J/m. In our simulations, we set the scaled temperature at $\tilde{A}=\frac{1}{4}$, corresponding to $\tilde{S}=\frac{3}{4}(1+\sqrt{1/3})$. The rotational diffusion, D*, is set to 0.35, equal to the value used in [17]. We varied the value of *d*_x_ to identify the behavior of the equilibrium configuration in the limit of *d_x_* → ∞. Our simulations are based on the initial conditions given in Section 2.2. We first focus on the strong anchoring conditions on the bounding plates and then on the weak anchoring conditions.

### 3.1. Strong Anchoring Boundary Conditions

#### 3.1.1. The Structure at Equilibrium State

**Figure 2.**
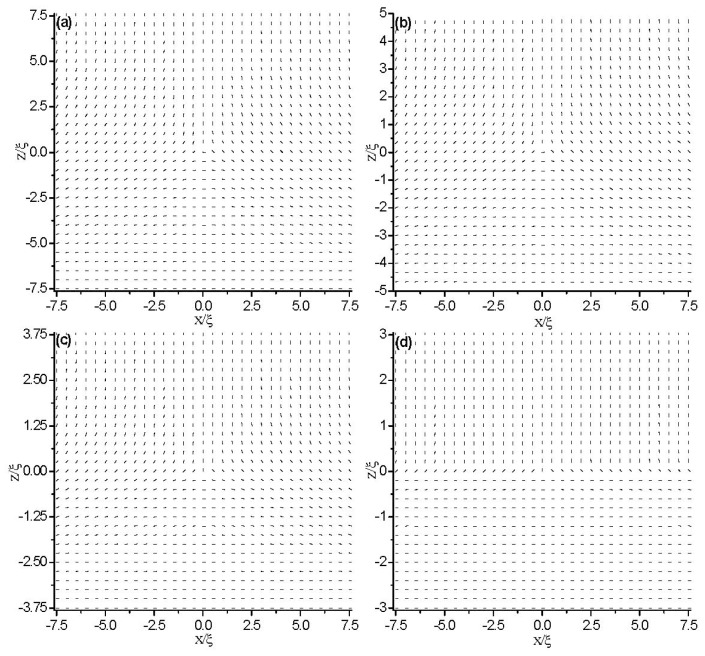
The director field profile at equilibrium state in a thin nematic layer with different thicknesses. The simulations are based on the initial conditions given in Section 2.2. (**a**) *d* = 15ξ; (**b**) *d* = 10ξ; (**c**) *d* = 7.5ξ; and (**d**) *d* = 6ξ.

**Figure 3.**
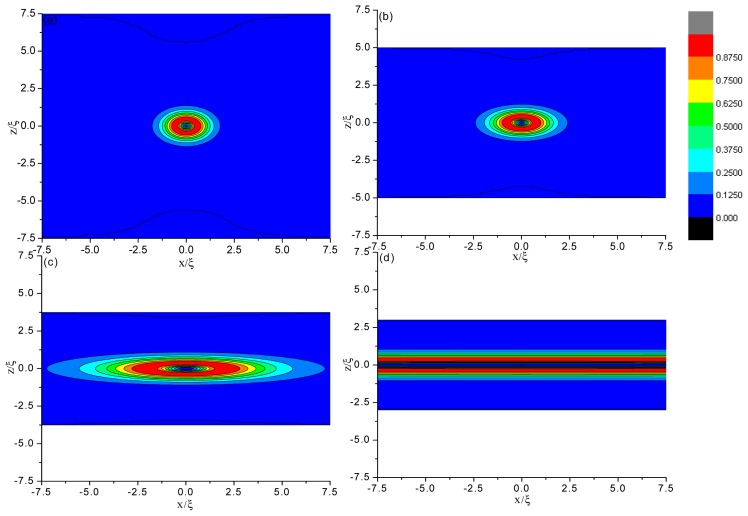
Biaxiality β^2^ for different thicknesses in a thin hybrid alignment nematic (HAN) layer with an initial line defect. (**a**) *d* = 15ξ; (**b**) *d* = 10ξ; (**c**) *d* = 7.5ξ; and (**d**) *d* = 6ξ.

Figures 2 and 3 show the director field profile and the calculated biaxiality, β^2^ (Equation (3)), inside the cell for different values of cell gap *d*. We find that at relatively large separations, the core structure is weakly influenced by *d* (see Figure 2a,b), and the nematic is uniaxial everywhere (β^2^ = 0), except for a small region around the defect core (Figure 3a,b). Its size is a few characteristic lengths, ξ. When the gap becomes comparable to ξ, the core of the defect explodes obviously along the *x* direction (Figure 2c) and a large biaxiality propagates inside of the cell (Figure 3c). The further decrease of the cell gap results in the creation of a biaxial wall, connecting the two orthogonal uniaxial directions imposed by the boundary conditions (Figures 2d and 3d) at a critical value of *d*_c_ ≈ 6ξ. In a word, the system has transited from the eigenvector rotation configuration with a topological defect into the eigenvalue exchange configuration by developing a thin transitional biaxial nematic layer.

**Figure 4.**
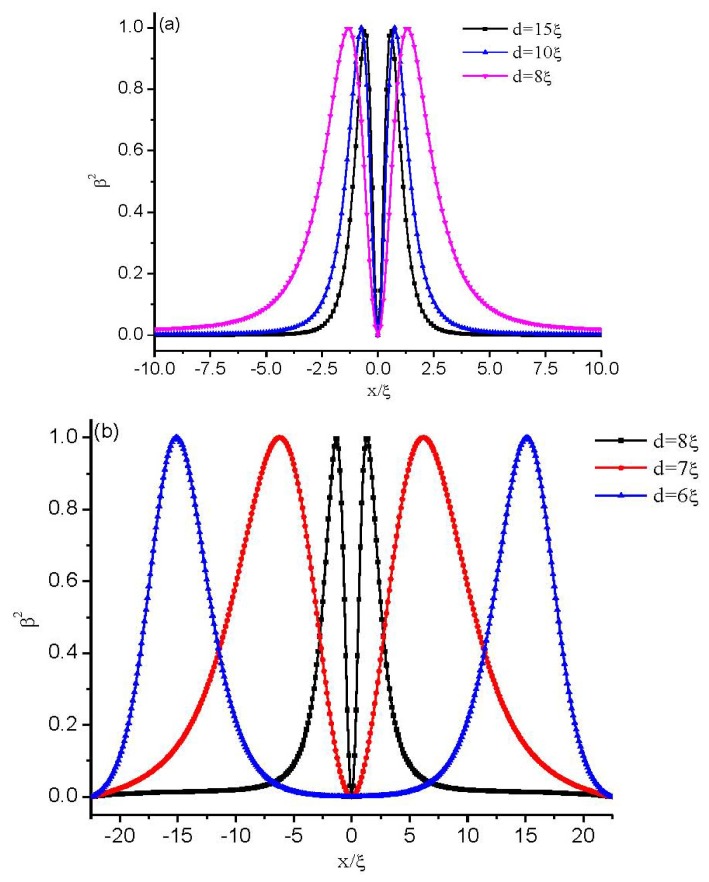
Biaxiality β^2^ across the defect center along *z* = 0 with different thicknesses. (**a**) Relatively large cell gaps; and (**b**) the cell gaps where the defect core explodes.

A more detailed analysis of the defect core, directly showing the process of biaxial transition, is given in Figure 4. There, we plot β^2^ across the defect center along *z* = 0 for different values of cell gap *d*. As the confining gap decreases, the distance at which β^2^ reaches its maximum value β^2^ = 1 (*i.e.*, the biaxial region) increased, indicating that the defect core becomes increasingly prolate in the *x* direction. By comparing the two figures in Figure 4, we see that at the cell gap where the defect core explodes, the changes of the biaxial region is faster.

**Figure 5.**
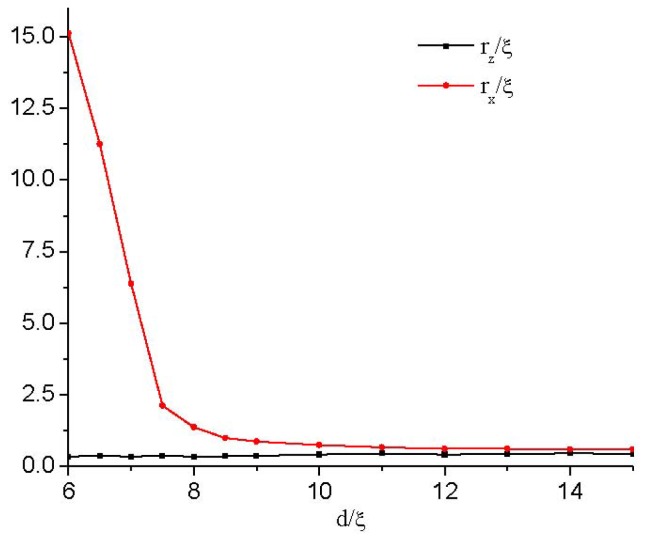
Variation of the defect core size with different thicknesses. The lengths of *r_x_* and *r_z_* estimate the core size along the *x*- and *z*-axes, respectively.

To quantify the influence of the cell gap on the defect structure, we plot in Figure 5 the change in both the width, *r_x_*, and height, *r_z_*, of the defect core as the cell gap, *d*, changes. We formally define *r_x_* and *r_z_* as the distance along the *x*- and *z*-axes, respectively, between the defect center and the point where β^2^ = 1. It is shown that *r_z_* is only slightly affected, while *r_x_* increases rapidly as the defect explodes. At a critical value of about *d* ≈ 6ξ, the value of *r_x_* diverges approximately, signaling a nematic order reconstruction. Therefore, the contours representing the biaxiality β^2^ become increasingly prolate in the *x* direction and, finally, into a plane (see Figure 3).

It seems that the transition to the biaxial wall originates from the biaxiality seed concentrated at the −1/2 defect. The biaxial wall would also be created in the absence of a defect [8,9]. According to [8,9], the two orthogonal uniaxial directors prescribed on the plate (HAN cell or TN cell with a π/2 twist) can be connected either through a director rotation or through an order reconstruction. When the cell gap is greater than a critical value *d* > *d*_c0_, the rotation solution is stable, while the exchange solution is unstable. When *d* < *d*_c0_, only the exchange solution exists as a stable configuration; no rotation solution exist. In other words, the rotation solutions merge continuously into the exchange solution at a critical value, *d*_c0_.

**Figure 6.**
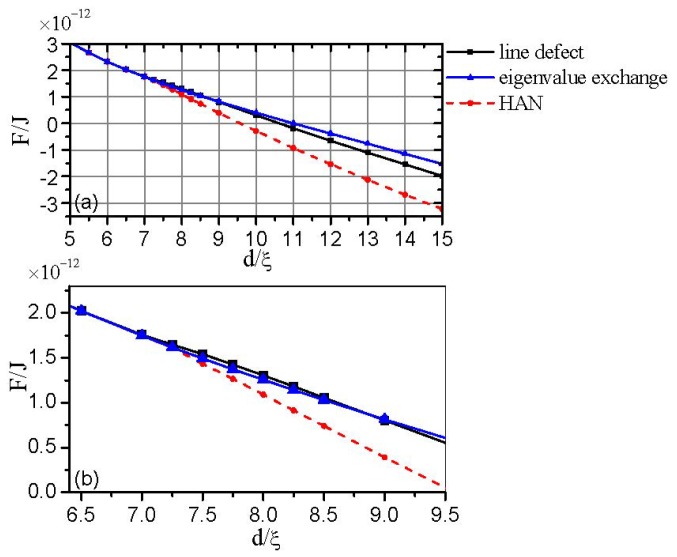
The free energy as a function of *d*/ξ for three different structures. (**b**) is a partial enlargement of (**a**).

In order to make the mechanism of the structure transition clear, we make a comparison between the two cases (with and without initial disclination). Figure 6 gives the free energy variation inside the cell for three structures (the structure with a defect core, eigenvalue exchange structure and HAN structure with no defect), where the free energy is calculated from numerical simulation results. Here, the HAN structure plays the role of a case of comparison.

Figure 6 shows that when the gap decreases to a critical value of *d*_c_* ≈ 9ξ, the exchange solution is stable, while the defect core solution is metastable. Comparing to the case with no initial disclination, we find that for the configuration with a topological defect, the value at which the exchange solution becomes stable increases relatively. That is because the configuration with a topological defect has larger energy, relative to the pure HAN configuration with no defect. With further decreasing of the cell gap until a critical value, *d*_c_, a transition of the defect core solution into the exchange solution happens. Below *d*_c_, only the exchange solution exists, but no defect core solution. According to the structure given by Figures 2 and 3, we conclude that for the case with initial disclinations, the value at which only the exchange solutions exist is about *d*_c_ ≈ 6ξ.

Figure 6b presents that at a value of *d*_c_*~* 7ξ, the two cases (with and without initial disclination) have nearly the same energy, while the simulated transition value with initial disclination is at *d*_c_*~* 6ξ. As mentioned in Section 2.2, we have prescribed fixed boundary conditions on the lateral walls, which will have an impact on the structure. In addition, there may be some calculation error. We speculate that for the two cases (with and without initial disclination), the values at which the structural transition takes place (only the exchange solutions exist) are almost the same, *i.e.*, *d*_c_ = *d*_c0_.

#### 3.1.2. Dynamical Evolution

**Figure 7.**
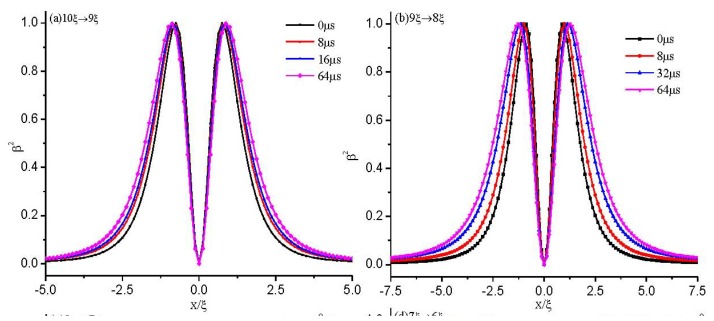

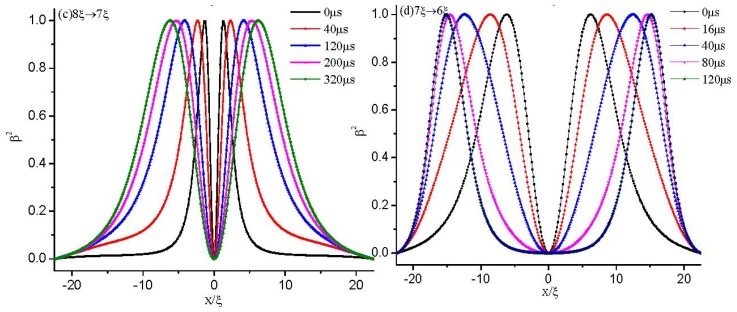
The dynamics of the biaxiality, β^2^, across the defect center along *z* = 0 for the structure with a defect. (**a**) 10ξ → 9ξ; (**b**) 9ξ → 8ξ; (**c**) 8ξ → 7ξ; and (**d**) 7ξ → 6ξ.

In order to make it clear how the configuration changes between two states, we make dynamical calculations of the defect core structure between different cell gaps (10ξ → 9ξ, 9ξ → 8ξ, 8ξ → 7ξ, 7ξ → 6ξ). Figure 7 shows the evolution process of the calculated biaxiality β^2^ across the defect center along *z* = 0 as the cell gap changes. It is shown that if the call gap, *d*, is greater than the critical value of *d*_c_, an initial structure with a defect core cannot change to the eigenvalue exchange structure, even at the value where the exchange structure is stable. The response time is about tens of microseconds, and the response becomes slower as the defect explodes (see Figure 7c).

**Figure 8.**
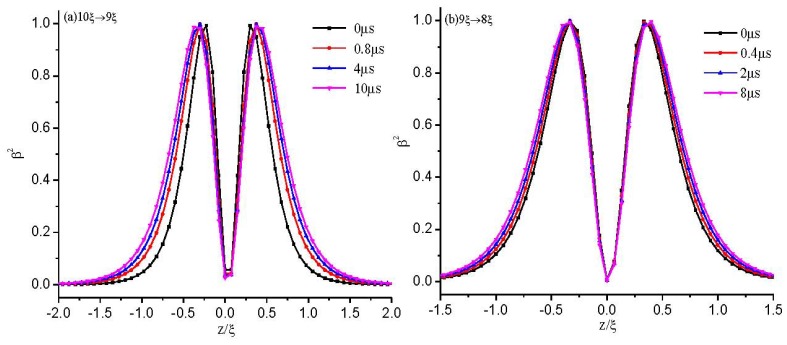

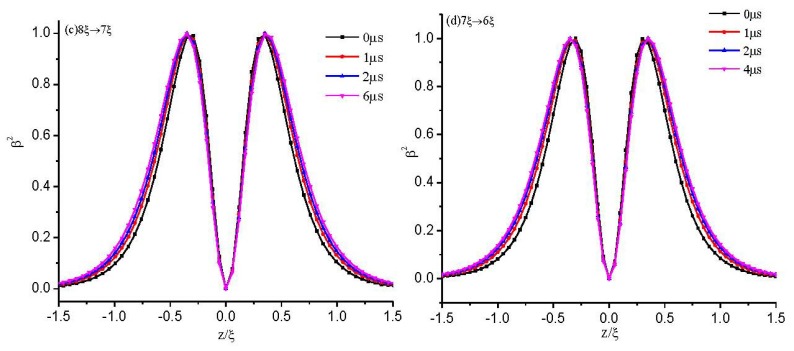
The dynamics of the biaxiality, β^2^, along *x* = 0 for the exchange solution. (**a**) 10ξ → 9ξ; (**b**) 9ξ → 8ξ; (**c**) 8ξ → 7ξ; and (**d**) 7ξ → 6ξ.

To analyze the influence of the defect core on the dynamic process, we make dynamical calculations of the exchange configurations between different cell gaps. To aid the comparisons, we give the dynamics as the cell gap decreases. Figure 8 shows the evolution process of the calculated biaxiality, β^2^, along *x* = 0 as the cell gap changes. It is shown that the response time is about several microseconds, and the response becomes faster as the cell gap decreases. Comparing Figure 7 with Figure 8, we find that the response between two exchange structures is much faster than that between two defect core structures.

Figures 7 and 8 show that above the critical value, *d*_c_, there are only defect-defect and exchange-exchange transitions, but no defect-exchange or exchange-defect transitions existing. However, the response of an initial defect configuration with *d* > *d*_c_ to *d* ≤ *d*_c_ (e.g., 7ξ → 6ξ) will undergo a defect-exchange transition (See Figure 7d). We expect that there are cell gap induced hysteresis effects, which are similar to hysteresis effects induced by the voltage in blue-phase liquid crystal [37]. As the cell gap, *d*, decreases, the defect core becomes increasingly prolate and undergoes a structural transition to the exchange configuration at a critical value of *d*_c_ ≈ 6ξ. While the cell gap, *d*, is increasing from *d <* 6ξ, the system is in the exchange configuration until *d* ≈ 9ξ, above which the system will change to the defect core configuration under the effect of thermal disturbance.

### 3.2. Weak Anchoring Boundary Conditions

**Figure 9.**
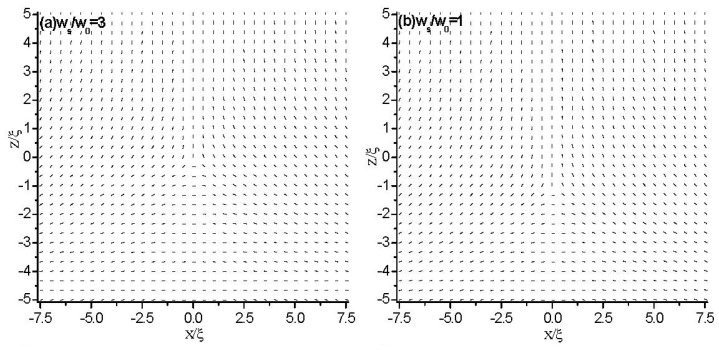

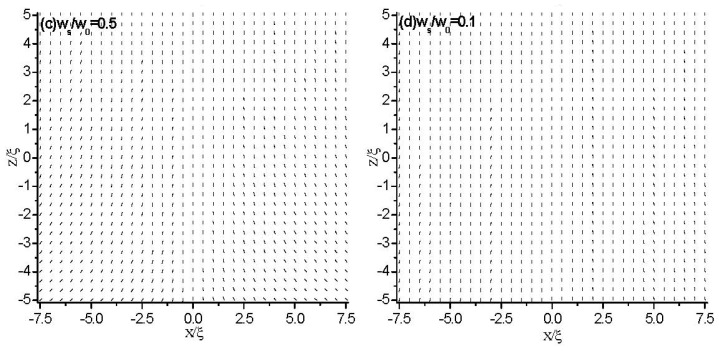
The director field profile at equilibrium state in a thin nematic layer with different anchoring strength on the bottom plate. The cell gap of the simulated nematic layer is *d* = 10ξ. (**a**) *W̃_s_=* 3; (**b**) *W̃_s_=* 1; (**c**) *W̃_s_=* 0.5; and (**d**) *W̃_s_=* 0.1.

In order to get the influence of the surface anchoring at the boundary plates on the behavior of topological defects, we focus on the Neumann conditions in this section. First, we study the condition of asymmetric boundaries. We impose strong homeotropic anchoring on the top plate and weak planar anchoring on the bottom plate. Figure 9 shows the director field profile inside a cell of thickness *d* = 10ξ for different values of *W̃_S_* at the bottom plate. It is shown that as the anchoring strength, *W̃_S_*, decreases, the defect glides towards the weak anchoring bottom plate and finally coalesces with the surface layer. This is consistent with the results in [38,39].

**Figure 10.**
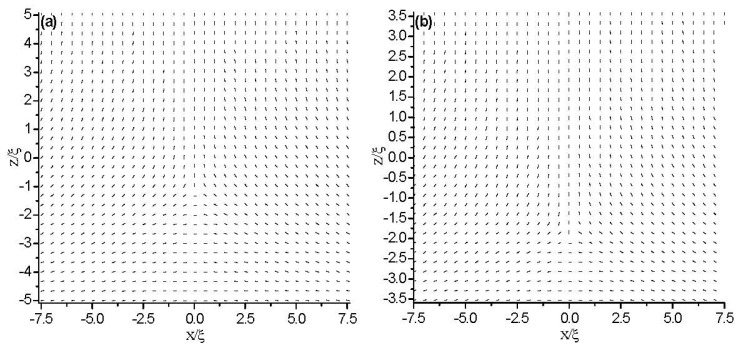

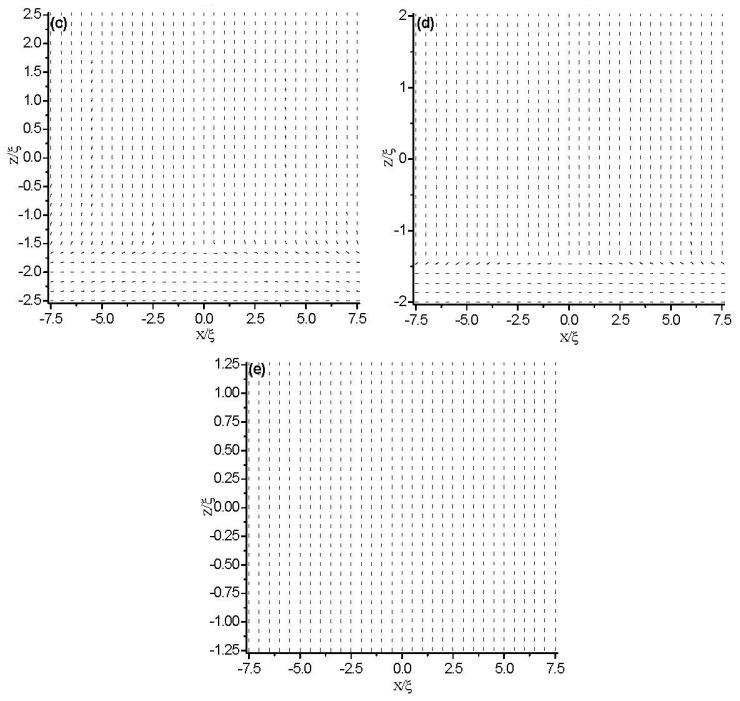
The director field profile at equilibrium state in a thin nematic layer with different thicknesses. The anchoring strength is *W̃_S_* = 1 at bottom plates. (**a**) *d* = 10ξ; (**b**) *d* = 7ξ; (**c**) *d* = 5ξ; (**d**) *d* = 4ξ; and (**e**) *d* = 2.5ξ.

**Figure 11.**
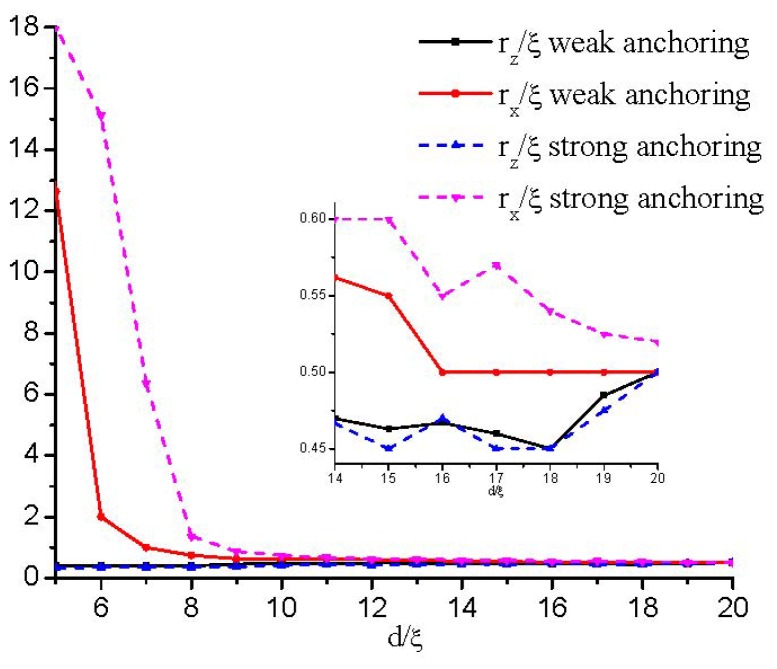
Variation of the defect core size with different thicknesses for the asymmetric boundaries. The lengths of *r_x_* and *r_z_* estimate the core size along the *x*- and *z*-axes, respectively. In the inset, the small region is enlarged. Here, the strong anchoring boundary plays the role of a case of comparison.

Next, we fix the anchoring strength at the bottom plate to *W̃_S_* = 1, to investigate the influence of the cell gap on the topological defects. Figures 10 and 11 show the director field profile, as well as the change in both the width *r*_x_ and height *r*_z_ of the defect core inside the cell for different values of cell gap *d*.

Figure 10 shows that the defect core is not located in the middle of the cell, which is due to the asymmetric boundary conditions. As the cell gap, *d*, decreases, the line defect moves towards the bottom plane. At a critical value *d*_c_(*w*_as_) ≈ 5ξ, the defect is transformed into a surface layer. The surface plane defect is then expelled from the cell when the cell gap reaches *d*_c_**(*w*_as_) ≈ 2.5ξ. For *d*_c_**(*w*_as_) < *d* < *d*_c_(*w*_as_), the surface biaxial layer bridges two uniaxial stares, one homeotropic in the bulk and the other planer on the boundary. The results are consistent with the results in [12,27].

Figure 11 shows that the change in *r*_z_ is slight, while *r*_x_ increases when the cell gap reaches a critical value, *d*_a_, where the defect core begins to lose its circular cross-section and stretches along the *x* direction. By comparing with the strong anchoring conditions (see the inset in Figure 11), we can conclude that the asymmetric boundary condition has a relatively smaller value of *d*_a_. That is because the moving of the defect as the cell gap decreases weakens the frustration on the confining surfaces. Figure 11 also gives that the value, *d*_c_(*w*_as_), where the defect is transformed into a surface layer, is smaller than that of strong anchoring conditions.

**Figure 12.**
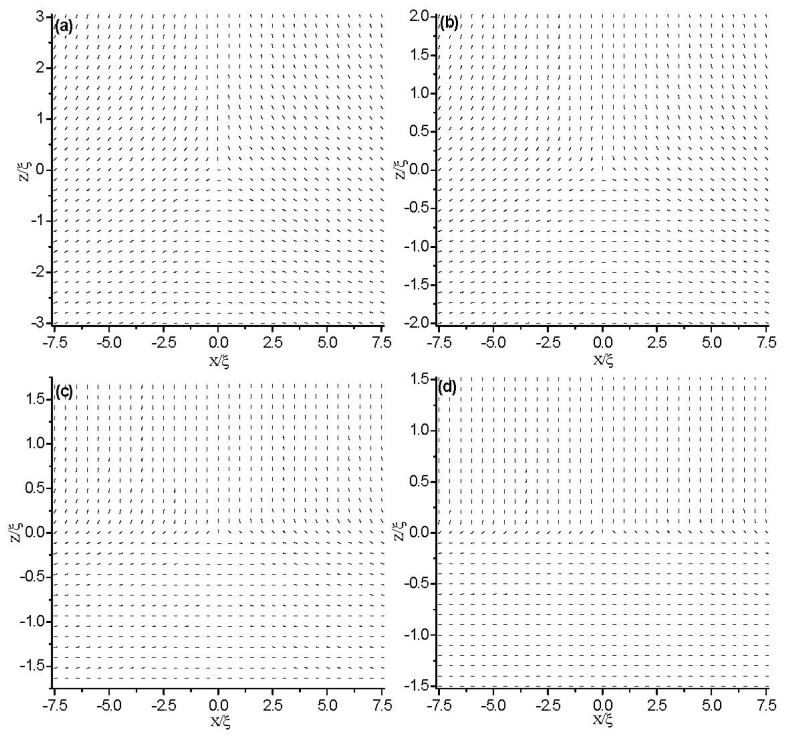
The director field profile at equilibrium state in a thin nematic layer with different thicknesses. The anchoring strength is *W̃_S_* =1 at both plates. (**a**) *d* = 6ξ; (**b**) *d* = 4ξ; (**c**) *d* = 3.5ξ; and (**d**) *d* = 3ξ.

Further, we study the condition of symmetric boundaries. We impose the same value of weak anchoring on both the two boundary plates and studied the effect of anchoring strength on the critical value, *d*_c_. In our simulation, the anchoring strength is set equal to *W̃_S_* = 1 at both plates. Figure 12 shows the director field profile inside the cell for different values of cell gap *d*, which shows that the structural transition takes place at a critical value of *d*_c_(*w*_s_) = 3ξ. Compared with the strong anchoring conditions (*i.e.*, Figure 2), we can conclude that weak anchoring boundary conditions decrease the critical value, *d*_c_. That is because the weak anchoring boundary weakens the frustration on the confining surfaces.

## 4. Conclusions

Within the Landau-de Gennes theory, we carried out a numerical study on the structure of *s* = −1/2 wedge disclinations and investigated the dynamics of biaxial transition in a nanoconfined HAN layer, using the two-dimensional finite-difference iterative method. Our results show that with the cell gap decreasing, the core of defect explodes and the biaxiality propagates inside the cell. Below a critical thickness, *d*_c_, of a HAN layer with a topological defect, an eigenvalue exchange transition occurs, and the defect is squashed flat into a biaxial layer. Because of the presence of the defect core, the value at which the exchange solution becomes stable increases relative to the pure HAN. The dynamic simulations show that before the critical value of *d*_c_, an initial structure with a defect core cannot change to the eigenvalue exchange structure, even at the value where the exchange structure is stable. The response time between two very close cell gaps is about tens of microseconds, and the response becomes slower as the defect explodes. For weak anchoring conditions, plates with strong anchoring repel the defects, while plates with weak anchoring allow the defect to escape through the boundary. Because of the weakened frustration, the weak anchoring boundary condition has relatively lower critical values of *d*_a_ and *d*_c_, corresponding to the value where the defect core begins to lose its circular cross-section and where the defect is transformed into a surface layer, respectively.

According to the parameters given, we can easily get the characteristic length ξ ~ 3.96 nm, and the critical separation is ~20 nm, which is consistent with the experimental result in [18]. Furthermore, the response time also agree with the experimental results (~10–100 μs) in [14]. The consistency of numerical calculation and experiment confirms the correctness and rationality of our theory, which further illustrates that the AFM can be used to carry on dynamics research.

Further, the parameters also give that the unit of anchoring strength is *W*_0_ ≈ 5.7 × 10^−4^ J/m^2^, which is reasonable. According to *b* = *k*/*W, W̃_s_*, = 0.1 corresponds to an extrapolation length of *b* ≈ 10^−7^ m, which is larger than the cell gap of *d* = 10ξ *~* 3.96 × 10^−8^ m, *i.e.*, *b*/*d* > 1. Figure 9 shows that at *W̃_s_* = 0.1, a sample of *d* = 10ξ presents a homeotropic orientation, which is consistent with the results of [39].

It can be predicted that the critical separation and the response time should be affected by the temperature; the detailed results, as well as the force curve with the *Q* tensor numerical model are tasks for the future.
